# MALAT1/miR‐185‐5p mediated high glucose‐induced oxidative stress, mitochondrial injury and cardiomyocyte apoptosis via the RhoA/ROCK pathway

**DOI:** 10.1111/jcmm.17835

**Published:** 2023-07-03

**Authors:** Ting Wang, Na Li, Lingling Yuan, Mengnan Zhao, Guizhi Li, Yanxia Chen, Hong Zhou

**Affiliations:** ^1^ Department of Endocrinology The Second Hospital of Hebei Medical University Shijiazhuang People's Republic of China

**Keywords:** cardiomyocyte apoptosis, diabetic cardiomyopathy, MALAT1, miR‐185‐5p, mitochondrial dynamics, RhoA

## Abstract

To explore the underlying mechanism of lncRNA MALAT1 in the pathogenesis of diabetic cardiomyopathy (DCM). DCM models were confirmed in db/db mice. MiRNAs in myocardium were detected by miRNA sequencing. The interactions of miR‐185‐5p with MALAT1 and RhoA were validated by dual‐luciferase reporter assays. Primary neonatal cardiomyocytes were cultured with 5.5 or 30 mmol/L D‐glucose (HG) in the presence or absence of MALAT1‐shRNA and fasudil, a ROCK inhibitor. MALAT1 and miR‐185‐5p expression were determined by real‐time quantitative PCR. The apoptotic cardiomyocytes were evaluated using flow cytometry and TUNEL staining. SOD activity and MDA contents were measured. The ROCK activity, phosphorylation of Drp1^S616^, mitofusin 2 and apoptosis‐related proteins were analysed by Western blotting. Mitochondrial membrane potential was examined by JC‐1. MALAT1 was significantly up‐regulated while miR‐185‐5p was down‐regulated in myocardium of db/db mice and HG‐induced cardiomyocytes. MALAT1 regulated RhoA/ROCK pathway via sponging miR‐185‐5p in cardiomyocytes in HG. Knockdown of MALAT1 and fasudil all inhibited HG‐induced oxidative stress, and alleviated imbalance of mitochondrial dynamics and mitochondrial dysfunction, accompanied by reduced cardiomyocyte apoptosis. MALAT1 activated the RhoA/ROCK pathway via sponging miR‐185‐5p and mediated HG‐induced oxidative stress, mitochondrial damage and apoptosis of cardiomyocytes in mice.

## 1 INTRODUCTION

Diabetic cardiomyopathy (DCM), a major complication of diabetes mellitus (DM), is characterized by diastolic dysfunction at its early stage and eventually leads to congestive heart failure (HF).^1^ DCM increases the risk of hospitalisation for HF and cardiovascular death in patients with DM. To date, therapy for DCM is based on the improvement of symptoms of HF. Despite extensive efforts, no specific treatment exists, which imply novel approaches are required. Hyperglycemia induces oxidative stress and mitochondrial damage in cardiomyocytes, which are considered as the critical process causing DCM.^2^, ^3^ Mitochondria play vital roles in pathophysiology of myocardium. The balance of dynamics between mitochondrial fission and fusion is very important for maintaining myocardial energy production and cardiomyocyte survival,^4^, ^5^ which are modulated by a series of dynamin‐related GTP proteins, including mitofusins (Mfn1/2) and dynamin‐related protein 1 (Drp1), etc. Emerging evidence has suggested that imbalanced mitochondrial dynamics contribute to the occurrence of DCM through mitochondrial oxidative stress and mitochondrial pathway‐mediated cardiomyocyte apoptosis.^6^, ^7^, ^8^ Abnormal morphology and dysfunction of mitochondria are usually observed in heart tissues in DCM.^9^ So, regaining mitochondrial homeostasis may be a potential therapeutic strategy for DCM.

Abundant evidence has demonstrated that epigenetic factors, such as non‐coding RNAs (ncRNAs), occupy an important position in the pathogenesis of DCM, including microRNAs (miRNAs) and long non‐coding RNAs (lncRNAs).^10^, ^11^, ^12^ MiRNAs are composed of approximately 22 nucleotide sequence, and regulate the expression of target genes at the post‐transcriptional levels via binding to the untranslated regions (UTRs) of complementary message RNAs.^13^ Several studies have verified that the differential expression of miRNAs in myocardium are involved in DCM in either patients or animal models with DM.^14^, ^15^, ^16^, ^17^ Recently, we screened the expression profiles of miRNAs in myocardium from DCM mice, and found that miR‐185‐5p, one of the differentially expressed miRNAs, was markedly down‐regulated in DCM mice (Figure 2A). The miRNA candidate target mRNAs were predicted by miRDB database (http://mirdb.org/miRDB/), and RhoA was shown to be a target gene of miR‐185‐5p, which were verified by NCBI (http://www.ncbi.nlm.nih.gov/). Our previous studies have shown that the RhoA/Rho kinase (ROCK) pathway in heart tissues is activated by hyperglycemia and involved in myocardial fibrosis in a rat model of Type 2 DM (T2DM), and inhibition of ROCK can improve the cardiac function of diabetic rats in vivo.^18^ In vitro, the RhoA/ROCK pathway mediates HG‐induced oxidative stress, cardiomyocyte apoptosis and fibroblast proliferation as well as collagen synthesis.^19^, ^20^ Plenty of evidence shows that the RhoA/ROCK pathway is closely related to mitochondrial dynamics.^21^, ^22^, ^23^

LncRNAs are non‐protein coding transcripts with more than 200 nucleotides, but they play crucial roles in multiple steps of gene regulation by serving as transcription cofactors, scaffolds for chromatin‐modifying complexes, molecular guides or decoys. LncRNAs act as competitive endogenous RNAs (ceRNAs) by sponging miRNAs, which change the expression of downstream target mRNAs.^11^, ^24^ Such, ceRNA networks consisting of lncRNA/miRNA/mRNA interactions play an important role in the pathological process of DCM.^25^, ^26^ LncRNA metastasis‐associated lung adenocarcinoma transcript 1 (MALAT1) is found to participate in the pathology of cardiomyocytes in response to diverse stimuli.^27^, ^28^, ^29^

In the present study, the interactions between MALAT1, miR‐185‐5p and RhoA were revealed by bioinformatics analysis. We would explore whether MALAT1, miR‐185‐5p and the RhoA/ROCK pathway play a role in HG‐induced mitochondrial damage and cardiomyocyte apoptosis, with the aim of providing a novel therapeutic target for DCM.

## 2 MATERIALS AND METHODS

### 2.1 Animals

Eight‐week‐old male db/db mice and C57BL/6J wild‐type mice were purchased from Nanjing Junke Biological Engineering Co., Ltd. The mice were housed in cages with room temperature (21–24°C), relative humidity (50%) and 12 h light–dark cycle. All mice were fed with standard chow and distilled water. Body weight, random bold glucose and systolic arterial blood pressure (SABP) were weekly measured. At the age of 22 weeks, cardiac function was assessed by cardiac ultrasound. Fasting blood samples were collected for further measurement of fasting blood glucose (FBG), fasting insulin (FINS), glycosylated haemoglobin (HbA1c), total cholesterol (TC), triglyceride (TG). The homeostasis model assessment for insulin resistance (HOMA‐IR) was calculated as FBG × FINS/22.5. The cardiac samples were extracted for subsequent experiments. Animal experiments were performed in accordance with the care and use of National Institutes of Health Guidelines and were approved by the Ethics Committee of the Second Hospital of Hebei Medical University.

### 2.2 Echocardiographic determination

At age of 22 weeks, mice were anaesthetized using 1.5% maintenance of isoflurane under continuous heart rate monitoring. All measurements were performed with a 11‐MHz linear transducer coupled to a high‐resolution Ultrasound System (vivid E95, GE Healthcare, USA). Fractional shortening (FS%) and ejection fraction (EF%) were used to assess cardiac systolic function. Peak velocity of early filling (E), atrial contraction (A) and early diastolic mitral valve flow velocity (e′) were obtained to evaluate cardiac diastolic function.

### 2.3 Transmission electron microscopy

Cardiac tissues were cut into fragments of 1 mm sections, which were fist fixed in 4% glutaraldehyde at 4°C overnight before being subjected to dehydration, soaking, embedding and staining. They were eventually cut into ultrathin sections of 50–70 nm, and the ultrastructure of myocardium was observed by transmission electron microscopy (TEM) (HT7800; Hitachi).

### 2.4 MiRNA sequencing

Left ventricular samples from three mice in each group were randomly selected for miRNA sequencing. The whole experiment process was performed by Kangcheng Biotechnology Co. Ltd. Briefly, total RNA of each sample was used for preparing the miRNA sequencing library. Then, the library was denatured as single‐stranded DNA, captured on Illumina flow cells, amplified in situ as clusters and subjected to 50‐cycle sequencing on Illumina NextSeq 500. Differentially expressed miRNAs were defined by a threshold with fold change ≥1.5 and *p* ≤ 0.05.

### 2.5 Culture of primary cardiomyocytesand treatment

All experimental procedures were approved by the Ethics Committee of the Second Hospital of Hebei Medical University. Cardiac tissues were acquired from 1 to 3 day old newborn C57BL/6J mice and the primary cardiomyocytes were obtained as previously described.^30^ When cardiomyocytes had been confirmed to reach 80% of confluence, they were randomized into experimental groups as follows: 5.5 mmol/L D‐glucose as the normal glucose (NG) group, osmolarity control (OSM) group containing 5.5 mmol/L D‐glucose plus 24.5 mmol/L mannitol and 30 mmol/L D‐glucose as the high glucose (HG) group. The myocytes were further incubated for 48 h in the presence or absence of MALAT1‐shRNA, NC‐shRNA (Genepharma) miR‐185‐5p mimics and miR‐185‐5p inhibitor as well as 100 μmol/L fasudil, a ROCK inhibitor, (Hongri). The concentration of fasudil 100 μmol/L was chosen according to our previous study.^20^ Cell transfection were performed using Lipofectamine™ 2000 (Invitrogen) according to the manufacturer's instructions.

### 2.6 RNA isolation and real‐time quantitative polymerase chain reaction (PCR)

Total RNA was extracted from cardiac samples using RNA‐easy™ Isolation Reagent (Vazyme). Then, total RNA was reverse‐transcribed into cDNA for lncRNA using Revert Aid First Strand cDNA Synthesis Kit (Thermo, USA) and subsequently for miRNA using miRNA OneScript™ cDNA Synthesis Kit (Vazyme). Real‐time quantitative PCR was conducted using GoTaq qPCR Master Mix (Promega). Gene expression was normalized to 18S rRNA or U6 through the 2^−ΔΔCT^ method. The sequences of primers were designed by Sangon Biotech Company as follows:

MALAT1: forward 5′‐GGGAGTGGTCTTAACAGGGAGGAG‐3′,

reverse 5′‐AACAGCATAGCAGTACACGCCTTC‐3′;

miR‐185‐5p: forward 5′‐CGCGTGGAGAGAAAGGCAGT‐3′,

reverse 5′‐AGTGCAGGGTCCGAGGTATT‐3′;

18S rRNA: forward 5′‐AGGGGAGAGCGGGTAAGAGA‐3′,

reverse 5′‐GGACAGGACTAGGCGGAACA‐3′;

U6: forward 5′‐CTCGCTTCGGCAGCACA‐3′,

reverse 5′‐AACGCTTCACGAATTTGCGT‐3′;

### 2.7 Dual‐luciferase reporter assay

The binding relations between miR‐185‐5p and MALAT1 or between miR‐185‐5p and RhoA were validated by dual‐luciferase reporter assays. The putative binding sites of miR‐185‐5p and MALAT1 or miR‐185‐5p and RhoA were determined through bioinformatics website Starbase (http://starbase.sysu.edu.cn/index.php) and miRDB (http://mirdb.org/miRDB/). Wild‐type (wt‐MALAT1 and wt‐RhoA) and mutant (mut‐MALAT1 and mut‐RhoA) sequences were designed and cloned into the pmirGlo Luciferase vectors (Promega, USA). HEK293T cells (Beyotime) were plated in 24‐well plates and incubated with the vectors to reach 80% confluence, and then were co‐transfected with miR‐185‐5p mimics or negative controls (GenePharma) for 48 h. whereafter, the luciferase activity was determined by using a Dual‐Lumi™ II Luciferase Kit (Beyotime).

### 2.8 Western blotting and pull‐down RhoA assay

Total proteins were obtained from the cell lysis buffer (Abcam) and were quantified using Bradford method. The proteins were separated by 10% sodium dodecyl sulfate‐polyacrylamide gel electrophoresis (SDS‐PAGE), and transferred to polyvinylidene fluoride (PVDF) membrane (Millipore). After sealed with 5% fat‐free milk at room temperature for 2 h, the membranes were added with primary antibodies at 4°C overnight: anti‐Bax, anti‐Bcl‐2, anti‐cleaved caspase‐3, anti‐Mfn2, anti‐p‐Drp1^S616^, anti‐β‐Tubulin (1:1000, respectively, Abways); anti‐p‐MYPT1, anti‐MYPT1 (1:1000 respectively, Cell Signalling). Then, the membranes were washed and incubated for 1 h at room temperature with secondary antibody labelled with horseradish peroxidase (HRP) (1:5000, Affinity). Finally, enhanced chemiluminescent (ECL) kits (Sharebio) were utilized to make the protein bands visualization, and the signal intensity of the interest proteins were analysed by ImageJ software. The level of individual protein was normalized by β‐Tubulin. The phosphorylation level of MYPT1 was standardized using total MYPT1.

RhoA activity was measured by the pull‐down RhoA assay according to the manufacturer's instructions. Cardiomyocytes were washed in TBS twice, and were lysed in ice‐cold lysis buffer. These lysates were centrifuged and incubated for overnight at 4°C with Rhotekin agarose to precipitate the GTP‐bound RhoA. These precipitates were washed and resuspended in 1 × loading buffer, then were boiled for 5 min. The total lysates and precipitates were analysed using Western blotting by a mouse monoclonal anti‐RhoA antibody (1:1000, Cytoskeleton).

### 2.9 Measurements of SOD activity and MDA content

The activity of superoxide dismutase (SOD) and the contents of malondialdehyde (MDA) in cellular supernatants were measured by commercial kits (Nanjing, China).

### 2.10 Flow cytometry

Cell apoptosis rate was examined using the Annexin V‐FITC/PI detection Kit (Neobioscience, China). Briefly, collected cardiomyocytes were resuspended in binding buffer and subsequently stained with Annexin V‐FITC and propidium iodide (PI) for 15 min at room temperature in the dark. Then, cell apoptosis rate was analysed by flow cytometry.

### 2.11 TdT‐mediated dUTP‐biotin nick end labelling (TUNEL) staining

Cell apoptosis assay (Beyotime) was performed by TUNEL staining according to the manufacturer's instructions. Briefly, cells were fixed with 4% paraformaldehyde in PBS for 30 min and permeabilized with 0.3% Triton X‐100 for 5 min at room temperature. Cells were labelled with TUNEL reaction mixture and were incubated at 37°C for 1 h and sealed with anti‐fluorescence quenching solution containing DAPI (Beyotime, China). TUNEL positive cells were observed with a fluorescence microscope (OLYMPUS, Japan).

### 2.12 Measurement of mitochondrial membrane potential (MMP)

The alteration in MMP was determined by JC‐1 assay kit (Beyotime). The cardiomyocytes were washed with Dulbecco's modified Eagle's medium (DMEM) and incubated with JC‐1 (1 μM) at 37°C for 20 min. After washing with DMEM, the samples were analysed by flow cytometry. The percentage of aggregated JC‐1 was used to quantify the change of MMP. A decreased percentage of aggregated JC‐1 represents the depolarization of the mitochondria, indicating a decrease in MMP.

### 2.13 Statistical analysis

GraphPad Prism 9.0 software (CA, USA) was used for statistical analyses. The experimental data were expressed as the mean ± SEM, with three independent experiments. Data were analysed using Student's *t* test between two groups and one‐way ANOVAs for more groups as well as followed with LSD test. A value of *p*<0.05 was considered statistically significant.

## 3 RESULTS

### 3.1 Db/db mice developed DCM

**FIGURE 1:**
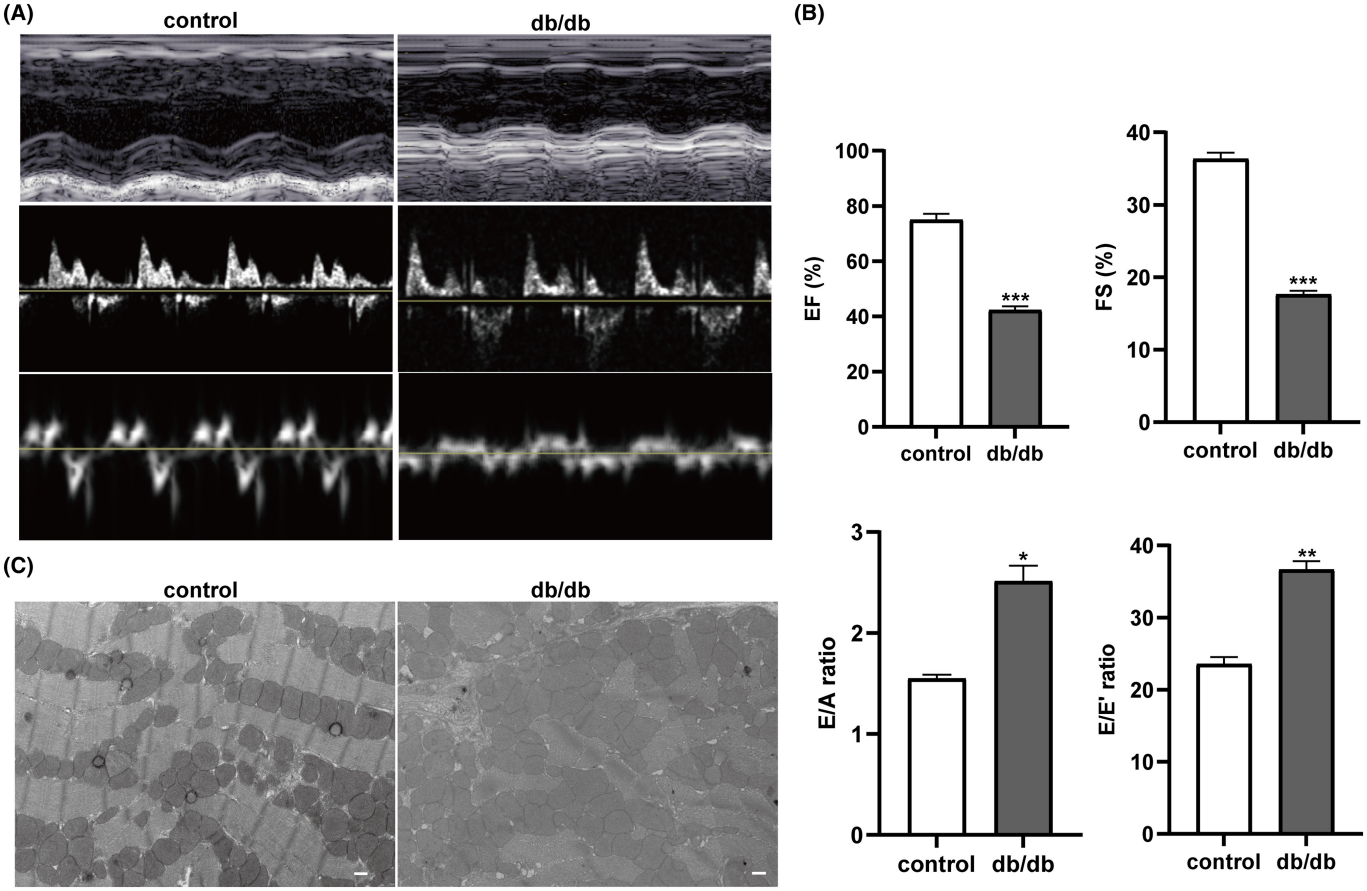
Alterations of cardiac function and structure in db/db mice at age of 22 weeks. (A) Representative pictures of M‐mode and tissue Doppler echocardiography. (B) Comparison of echocardiographic indices of cardiac systolic and diastolic function between db/db mice and control mice. (C) Ultrastructural alterations in myocardium detected by TEM (×3000, scale bar = 5.0 μm). *n* = 3. *p < 0.05, **p < 0.01, ***p < 0.001 versus control mice. EF, Ejection fraction; FS, Fractional shortening; E/A, ratio of E wave to A wave; E/e′, ratio of E wave to e′ wave.

Metabolic parameters and the ratio of heart weight to body weight (HW/BW) at age of 22 weeks are shown in Table 1. Compared with control mice, FBG, FINS, HbA1c, HOMA‐IR and HW/BW were remarkably increased in db/db mice (*p*<0.001, *p*<0.01, respectively). There was no significant difference in SABP between db/db and control mice. Representative pictures of echocardiography are presented in Figure 1A. Compared with control mice, the EF% and the FS% were significantly decreased while the E/A and the E/e′ were enhanced in db/db mice (Figure 1B). The results revealed that the cardiac systolic as well as diastolic function were impaired in db/db mice. Myocardium ultrastructure was visualized in Figure 1C. Obvious mitochondria swelling, cristae disorder and myofibril fracture were observed in myocardium of db/db mice.

**TABLE 1:** Metabolic parameters in db/db mice (*N* = 6) at the age of 22 weeks.

General parameters	Control mice	db/db mice
FBG (mmol/L)	5.64 ± 0.24	32.81 ± 1.23***
FINS (mU/L)	77.76 ± 5.83	312.18 ± 19.09***
HbA1c (%)	4.50 ± 0.05	9.13 ± 0.44***
HOMA‐IR	19.55 ± 1.80	453.54 ± 36.46***
TG (mmol/L)	1.07 ± 0.03	1.59 ± 0.25
TC (mmol/L)	2.56 ± 0.10	4.20 ± 0.31***
HW/BW (×10^−3^)	4.61 ± 0.18	5.25 ± 0.11**

*Note*: All data shown are mean ± SEM for three independent experiments with Student's *t‐*test.

Abbreviations: FBG, fasting blood glucose; FINS, fasting insulin; HbA1c, haemoglobin A1c; HOMA‐IR, homeostasis model assessment for insulin resistance, is calculated as FBG (mmol/L) × FINS (mU/L)/22.5; HW/BW, ratio of heart weight/body weight; TG, triglyceride; TC, total cholesterol.

** *p* < 0.01

*** *p* < 0.001 versus control mice.

### 3.2 Upregulated MALAT1 and downregulated miR‐185‐5p in db/db mice

**FIGURE 2:**
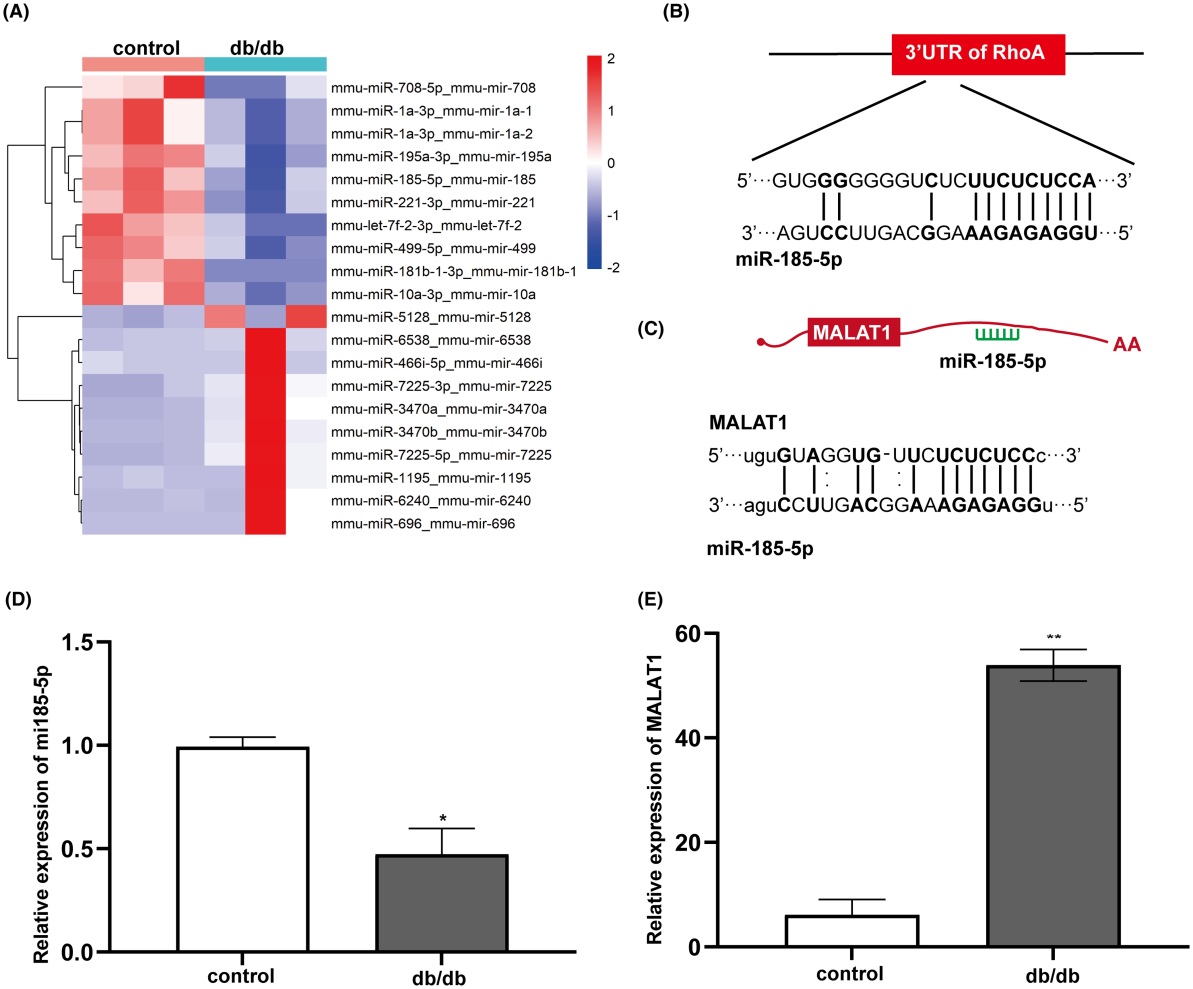
The expression characteristics of MALAT1 and miR‐185‐5p in db/db mice. (A) The top 10 up‐regulated and down‐regulated miRNAs were shown by Heatmap. Red colour represents high relative expression level and blue represents low relative expression level. (B) The complementary sequence between miR‐185‐5p and RhoA was forecasted by mirdb. (C) The binding sites between MALAT1 and miR‐185‐5p were predicted by Starbase. (D, E) Expression of miR‐185‐5p and MALAT1 in cardiac tissues by real‐time quantitative PCR. Data represent the means ± SEM (*n* = 3). *p < 0.05, **p < 0.01 versus control mice.

The analysis of miRNA sequencing revealed significant changes in the miRNA expression profile in db/db mice compared with control mice. The top 10 up‐regulated and down‐regulated miRNAs were shown by Heatmap (Figure 2A). Among top 10 dramatically downregulated miRNAs, miR‐185‐5p was shown to target the RhoA/ROCK pathway by using database of miRDB (http://mirdb.org/miRDB/), which were verified by NCBI (http://www.ncbi.nlm.nih.gov/). So, RhoA may be a target gene of miR‐185‐5p. According to ceRNA network and bioinformatics website Starbase (http://starbase.sysu.edu.cn/index.php), MALAT1 maybe acts as a ceRNA for miR‐185‐5p (Figure 2B,C). The expression of miR‐185‐5p was significantly downregulated in heart tissues in db/db mice by real‐time quantitative PCR, consistent with the results of miRNA sequencing, while the expression of MALAT1 in db/db mice was significantly upregulated (Figure 2D,E).

### 3.3 MALAT1 activated the RhoA/ROCK pathway via sponging miR‐185‐5p in cardiomyocytes in HG condition

**FIGURE 3:**
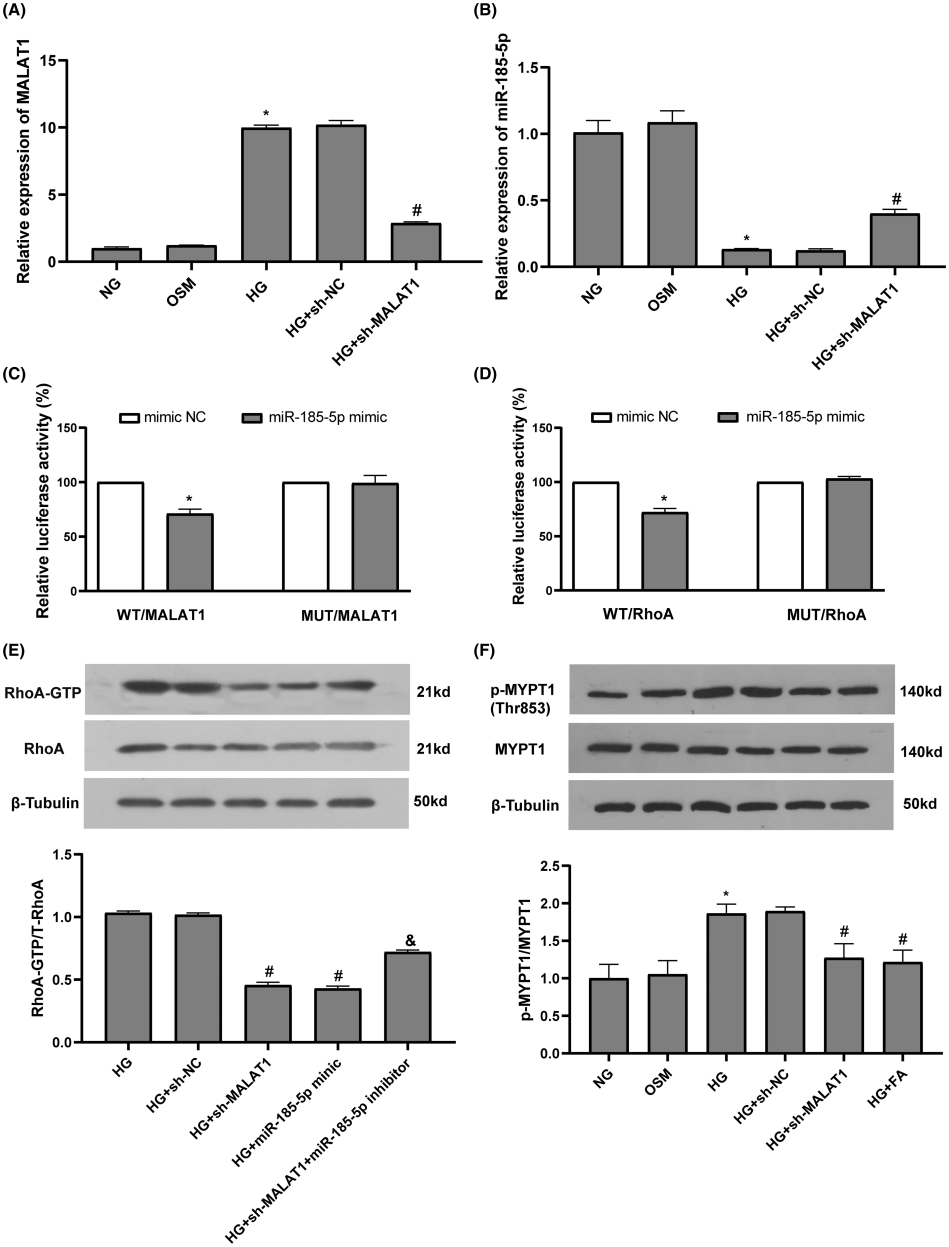
MALAT1 activated the RhoA/ROCK pathway via sponging miR‐185‐5p in cardiomyocytes in HG. (A, B) The expression of MALAT1 and miR‐185‐5p in cardiomyocytes were determined by real‐time quantitative PCR. (C, D) The interactions between miR‐185‐5p and MALAT1 or RhoA were validated by the dual‐luciferase reporter assay. (E) The interactions between miR‐185‐5p and MALAT1 or RhoA were proved by the rescue experiment. (F) The phosphorylation of MYPT1 were evaluated by Western blotting. Data represent the means ± SEM (*n* = 3). *p < 0.05 versus NG group; #*p* < 0.05 versus HG group; &*p* < 0.05 versus HG + sh‐MALAT1 group. FA, fasudil; HG, high glucose; NG, normal glucose; OSM, osmolarity control.

Compared with the NG group, exposure of cardiomyocytes to HG significantly increased the expression of MALAT1 and decreased the expression of miR‐185‐5p. Compared with the HG group, MALAT1‐shRNA significantly decreased the expression of MALAT1 and increased the expression of miR‐185‐5p in cardiomyocytes exposed to HG (Figure 3A,B). Bioinformatic analysis predicts that there are binding sites between miR‐185‐5p and MALAT1 or RhoA (Figure 2B,C). The dual‐luciferase reporter assay showed that transfection with miR‐185‐5p mimics markedly reduced the luciferase activity of wt‐MALAT1; however, mut‐MALAT1 luciferase activity had no changes (Figure 3C). Transfection with miR‐185‐5p mimics markedly decreased the luciferase activity of wt‐RhoA, while mut‐RhoA luciferase activity was not affected (Figure 3D). Compared with the HG group, MALAT1‐shRNA dramatically decreased RhoA activity in cardiomyocytes exposed to HG, which were reversed by addition of miR‐185‐5p inhibitor (Figure 3E). These results show that miR‐185‐5p could directly bind to MALAT1 and RhoA, MALAT1 sponges miR‐185‐5p to regulate RhoA activity in cardiomyocytes.

MYPT1 is the major effector of ROCK and the phosphorylation of MYPT1 represents ROCK activity. Compared with the NG group, HG stimulated ROCK activity in cardiomyocytes. Compared with the HG group, MALAT1‐shRNA and fasudil markedly decreased ROCK activity in cardiomyocytes exposed to HG (Figure 3F).

### 3.4 MALAT1 and the RhoA/ROCK pathway mediated oxidative stress, mitochondrial injury and cardiomyocyte apoptosis in HG condition

**FIGURE 4:**
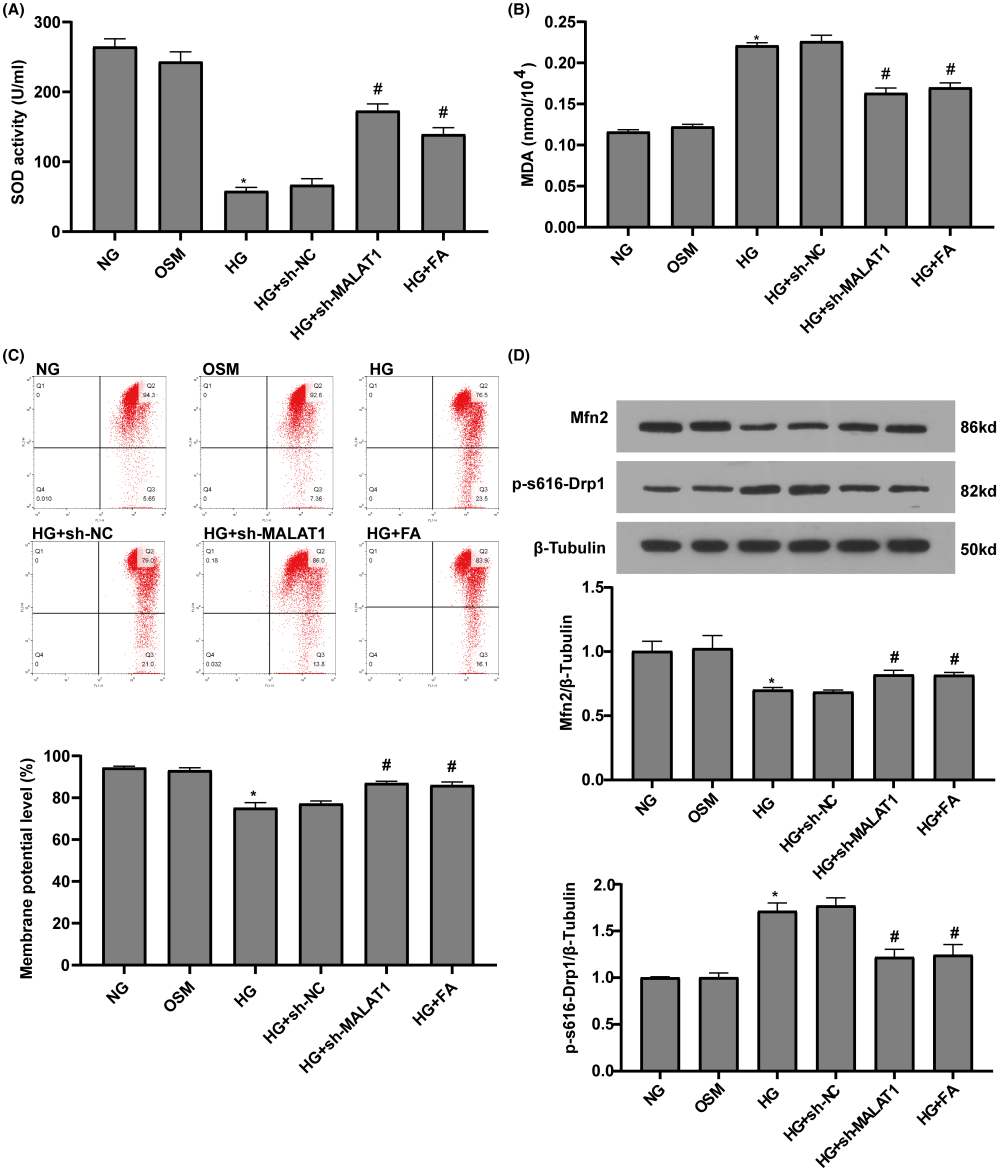
Knockdown of MALAT1 and fasudil inhibited HG‐induced oxidative stress and mitochondrial injury. (A, B) MDA contents and SOD activity in the cellular supernatants were measured. (C) Mitochondrial membrane potential in cardiomyocytes was evaluated by JC‐1. (D) The levels of Mfn2 protein and phosphorylation of Drp1^S616^ were measured by Western blotting. Data represent the means ± SEM (*n* = 3). *p < 0.05 versus NG group; #*p* < 0.05 versus HG group. FA, fasudil; HG, high glucose; NG, normal glucose; OSM, osmolarity control.

We investigated oxidative stress in the cellular supernatants by the content of MDA, a classic oxidative damage marker and the activity of SOD, an antioxidant marker. Compared with NG group, MDA content was significantly increased while SOD activity was significantly decreased in the HG group. Treatments of cardiomyocytes with MALAT1‐shRNA and fasudil markedly decreased HG‐induced increase in MDA content and enhanced SOD activity. There were no significant differences in MDA content and SOD activity between the OSM and NG groups (Figure 4A,B).

As shown in Figure 4C, remarkably decreased MMP level was observed in cardiomyocytes exposed to HG, which were improved by MALAT1‐shRNA and fasudil. The balance of mitochondrial fission and fusion is very important for keeping normal mitochondrial function. Compared with NG group, exposure of cardiomyocytes to HG significantly decreased Mfn2 protein level, while increased the phosphorylation of Drp1 at S616, a key signal activating mitochondrial fission. MALAT1‐shRNA and fasudil effectively reversed these changes induced by HG (Figure 4D). There were no significant differences in the levels of MMP, Mfn2 and the phosphorylation of Drp1^S616^ between the OSM and NG groups.

### 3.5 MALAT1 and the RhoA/ROCK pathway mediated cardiomyocyte apoptosis in HG condition

**FIGURE 5:**
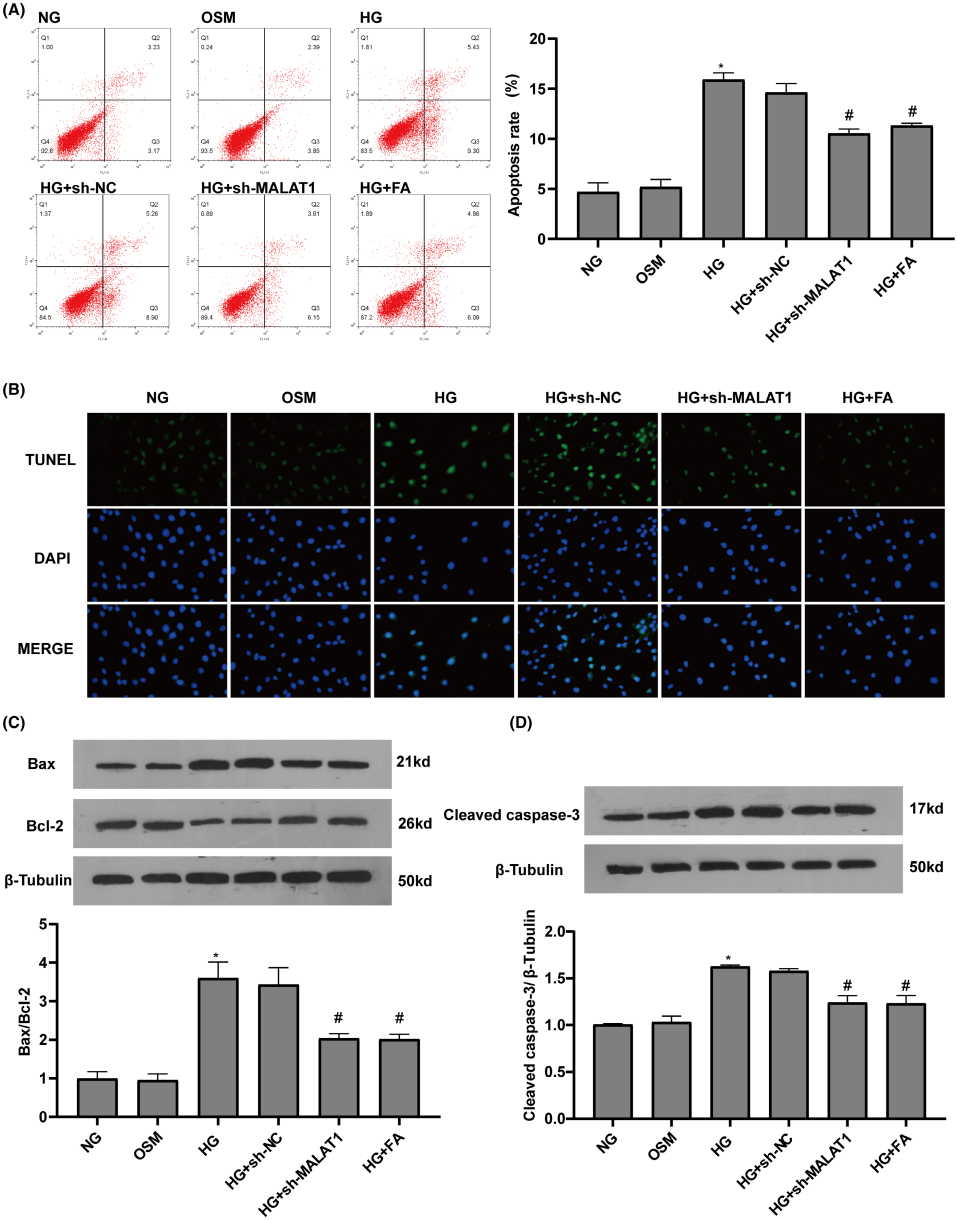
Knockdown of MALAT1 and fasudil inhibited HG‐induced cardiomyocyte apoptosis. (A, B) Cardiomyocyte apoptosis was evaluated by flow cytometry and TUNEL staining. (C, D) The Bax/Bcl‐2 protein levels and cleaved caspase‐3 protein expression were measured by Western blotting. Data represent the means ± SEM (*n* = 3). *p < 0.05 versus NG group; #*p* < 0.05 versus HG group. FA, fasudil; HG, high glucose; NG, normal glucose; OSM, osmolarity control.

Cardiomyocyte apoptosis is shown in Figure 5A,B, dramatically increased cell apoptosis was observed in the presence of HG. MALAT1‐shRNA and fasudil treatment significantly suppressed HG‐induced increase of cardiomyocyte apoptosis. It is well known that the occurrence of cell apoptosis is regulated by apoptosis‐associated proteins. Compared with the NG group, the ratio of Bax to Bcl‐2 protein (Bax/Bcl‐2) and cleaved caspase‐3 levels were significantly increased cardiomyocytes exposed to HG, which were counteracted by MALAT1‐shRNA and fasudil (Figure 5C,D). There were no significant differences in cell apoptosis and apoptosis‐associated proteins between the OSM and NG groups, suggesting that HG‐induced cardiomyocyte apoptosis was independent of high osmotic pressure.

## 4 DISCUSSION

Db/db mice are classically used as animal models of T2DM. In this study, db/db mice exhibited hyperglycemia and insulin resistance, which are in accord with the characteristics of T2DM. The results of echocardiography and TEM showed cardiac dysfunction and abnormal myocardium ultrastructure, suggesting that db/db mice developed DCM. Recently, the roles of lncRNAs and miRNAs in the development of DCM have been given more and more attention.^31^, ^32^ MiRNAs play a crucial role in many biological processes, such as cell proliferation, differentiation and apoptosis, through the suppression of target gene expression. It is reported that a few of differential miRNAs in hearts are implicated in oxidative stress, inflammatory processes, cardiomyocyte pyroptosis and apoptosis in DCM.^31^ LncRNAs have many biological functions, including action as scaffolds, chromatin modification and regulation of gene expression. In particular, lncRNAs can act as miRNA sponges to influence the regulatory actions of miRNAs by binding to them to block the interactions with their target genes.^33^ Some studies have indicated the essential roles of lncRNA‐miRNA‐mRNA interaction network in the pathomechanisms of DCM.^34^, ^35^ Our study showed that miR‐185‐5p was one of the top 10 downregulated miRNAs in myocardium of DCM mice by miRNA sequencing, consistent with the results of PCR. Overexpression of MALAT1 in myocardium of DCM mice was also confirmed by PCR. According to bioinformatic analysis, there are the binding sites of miR‐185‐5p to MALAT1 and RhoA. Hence, it is necessary to further identify the interactions of miR‐185‐5p with MALAT1 and RhoA as well as their roles in HG‐induced oxidative stress, mitochondrial injury and cardiomyocyte apoptosis in vitro.

Collecting evidence shows that oxidative stress plays a crucial role in development of DCM.^36^, ^37^ Glucose overload in mitochondria leads to excessive reactive oxygen species (ROS) generation and mitochondrial dysfunction, which in turn enhance oxidative stress.^38^ Enhanced oxidative stress is closely associated with the induction of cell apoptosis.^39^, ^40^ Cardiomyocyte apoptosis is key step triggering the progression of DCM, which subsequently result in the remodelling and fibrosis of the myocardium, ultimately leading to impaired myocardial function.^41^ In the study, we observed increased MDA and reduced SOD in the medium in HG, concurrently increased apoptosis of cardiomyocytes, Bax/Bcl‐2 and cleaved caspase‐3, meaning that HG induced oxidative stress and triggered mitochondrial pathway dependent cardiomyocyte apoptosis. Mitochondrial dynamics not only determine mitochondrial morphology but also regulate mitochondrial function, including energy production, oxidative stress and cell apoptosis.^9^, ^42^ Mfn2 modulates mitochondrial fusion, and phosphorylation of Drp1^S616^ activates mitochondrial fission while phosphorylation of Drp1^S637^ has the inhibitory effect on fission. Only Mfn2 and Drp1 work in harmony, the homeostasis of mitochondrial dynamics is sustained. Our study showed downregulated Mfn2 protein and increased phosphorylation of Drp1^S616^ in cardiomyocytes exposed to HG, accompanied by decreased MMP. Similarly, anomalies in mitochondrial morphology were observed in the myocardium of DCM mice. These results suggest that impaired mitochondrial fusion and excessive mitochondrial fission induced by HG are implicated in mitochondrial damage. More and more studies have revealed that the imbalanced mitochondrial dynamics contributes to the development of DCM, and that targeting mitochondrial dynamics is a novel strategy of therapy for DCM.^6^, ^8^, ^43^, ^44^

MALAT1 is one of abundantly expressed lncRNAs in cardiac myocytes and highly conserved in mammals.^45^, ^46^ The studies show that MALAT1 is involved in the development and progression of diabetic complications, and would be a novel target of diagnosis and treatment for these complications.^47^, ^48^ It has been reported that MALAT1 was up‐regulated in hearts of diabetic rats, and knockdown of MALAT1 significantly attenuated inflammation and cardiomyocyte apoptosis and improved diabetes‐induced cardiac dysfunction.^27^, ^49^ MALAT1 recruited histone methyltransferase EZH2 to the promoter region of miR‐22 to inhibit the transcription of miR‐22, thereby leading to cardiomyocyte apoptosis in DCM.^50^ These results are in agreement with our findings that MALAT1 was upregulated in either HG‐treated cardiomyocytes in vitro or the hearts of DCM mice in vivo, and knockdown of MALAT1 inhibited HG‐induced oxidative stress, mitochondrial injury and cardiomyocyte apoptosis. Recent studies have revealed that dysregulated lncRNA‐mRNA network based on ceRNAs is involved in the pathological process of myocardium.^51^, ^52^ The expression of miR‐185‐5p was decreased in hypoxia/reoxygenation (H/R) treated human cardiac myocytes (HCMs), and negatively regulated by lncRNA ROR, ROR/miR‐185‐5p/CDK6 axis modulates HCM pyroptosis and inflammatory response induced by H/R.^53^ MALAT1 has been found to act as a ceRNA for miR‐185‐5p in lung cancer,^54^ and RhoA is a target gene of miR‐185 in human colorectal cells.^55^ Similarly, we affirmed that the mutual effects of miR‐185‐5p on MALAT1 and RhoA by dual‐luciferase reporter assay. HG significantly increased MALAT1 expression as well as RhoA and ROCK activity, and concurrently reduced miR‐185‐5p expression. Knockdown of MALAT1 led to increased miR‐185‐5p expression as well as decreased RhoA and ROCK activity. The results of rescue experiment further manifested that MALAT1 acted as a ceRNA for miR‐185‐5p to regulate RhoA activity. Such, overexpression of MALAT1 in cardiomyocytes in HG condition activated the RhoA/ROCK pathway via sponging miR‐185‐5p. Knockdown of MALAT1 or inhibition of ROCK by fasudil all alleviated HG‐induced oxidative stress, mitochondrial damage and cardiomyocyte apoptosis. Our previous study has also confirmed that the RhoA/ROCK pathway plays a crucial role in HG‐induced oxidative stress and cardiomyocyte apoptosis.^20^ Increases in oxidative stress and cardiomyocyte apoptosis as well as impaired mitochondria all contribute to the development and progression of DCM.^10^, ^56^

Due to highly conservative property and stability of lncRNAs, lncRNAs have been considered as potential targets of diagnosis and therapy for DCM. It is possible to ameliorate DCM through overexpression of protective lncRNAs and knockdown of detrimental lncRNAs in the hearts. Our study showed that MALAT1 activated RhoA/ROCK pathway via sponging miR‐185‐5p and mediated HG‐induced oxidative stress, mitochondrial damage and apoptosis in cardiomyocytes, which indicate that MALAT1 is a vital regulator of DCM and acts as a promising target of diagnosis and therapy for DCM.

## AUTHOR CONTRIBUTIONS

**Ting Wang:** Investigation (lead); methodology (lead); writing – original draft (lead). **Na Li:** Resources (lead). **Lingling Yuan:** Resources (equal). **Mengnan Zhao:** Software (equal). **Guizhi Li:** Software (equal). **Yanxia Chen:** Validation (equal). **Hong Zhou:** Conceptualization (lead); project administration (lead); supervision (lead); writing – review and editing (lead).

## CONFLICT OF INTEREST STATEMENT

The authors have no conflict of interest to disclose.

## Data Availability

The data that support the findings of this study are available from the corresponding author upon reasonable request.
